# Supplementary material for: Effect of PCSK9 inhibition with evolocumab on lipoprotein subfractions in familial dysbetalipoproteinemia (type III hyperlipidemia)
Source: PLoS One. 2022 Mar 23;17(3):e0265838. doi: 10.1371/journal.pone.0265838 (PMC8942248; doi:10.1371/journal.pone.0265838)
Supplement: Attachment — Submitted filename: Response to Reviewers R2.pdf [file pone.0265838.s002.pdf]

Response to Reviewers:

Reviewer #1:

No further comments

Reviewer #2:

Almost all comments have been addressed. Sample size remains a major issue that cannot be resolved.

Minor comments:

Please revise abbreviations and typos (e.g. Celcius, PCSK-9 or PCSK9)

Thank you for pointing out these mistakes. They were corrected in the current version of the manuscript.

Reviewer #3:

Dear author my major concearn is the lack of an accurate definition of the patients included in the study.

In fact, I am awared that familial dysbetalipoproteinemia is rare but for this reason, patients should be very well characterized. Similarly, the "control" population lack of definition as for example, you have included patients with FH with those suffering with other form of hypercholesterolemia in a unique group. I believe that these could be potentially confounders. I suggest publishing this data as a short communication rather than as an original article. The study is interesting but some consideration has to be done.

Thank you for suggesting to better characterize the subjects. We have now added 3 paragraphs to the method section in which we describe the selected patients in more detail. Although we understand that the underlying pathophysiology is important, the effect of evolocumab on the lipid profile seems to depend mostly on the phenotype (and on the availability of functional LDL receptors).

We did not change the submission to a short report as this would counteract the suggestion of the reviewer to describe the patient groups in more detail.
