# Supplementary material for: Effect of PCSK9 inhibition with evolocumab on lipoprotein subfractions in familial dysbetalipoproteinemia (type III hyperlipidemia)
Source: PLoS One. 2022 Mar 23;17(3):e0265838. doi: 10.1371/journal.pone.0265838 (PMC8942248; doi:10.1371/journal.pone.0265838)
Supplement: Attachment — Submitted filename: Response to Reviewers.docx [file pone.0265838.s001.docx]

**Response to Reviewers:**

We thank the reviewers for their thoughtful and constructive comments. We have incorporated them into the revised version and believe that this has considerably improved the manuscript. We hope that the manuscript will now be fond acceptable for publication.

**Reviewer #1:** Although I believe the study is interesting, it contains some issues that need to be sorted out.

The introduction misses the part relative to PCSK9, e.g., the role of PCSK9 on the expression of LDL-R, and the general impact of PCSK9 inhibition in terms of LDL-C lowering and atherosclerotic cardiovascualr disease (ASCVD) risk reduction. Concerning the biology of PCSK9, the Authors can refer to a recent review (PMID: 34019847). A similar comment is valid in the case of lipoprotein(a). Relative to this, the discussion lacks to mention the impact of PCSK9 inhibition on lipoprotein(a). Moreover, lipoprotein(a) levels seem to be very high and in the hypercholesterolemic group, they overtake the ASCVD risk threshold of 50 mg/dL. The Authors should evaluate the contribution of cholesterol carried by lipoprotein(a), and calculate, in addition, the corrected LDL-C (PMID: 34450317). Considering that, for lipoprotein(a), conversion between mg/dL and mmol/L is not advisable, cholesterol should be expressed both in mmol/L and mg/dL. Please report the assay that has been used to measure lipoprotein(a).

We thank the reviewer for this thoughtful comments. We have addressed these topics in the revised version of the manuscript and also included the suggested references. Specifically, in the introduction we have extended the section on PCSK9 inhibition, stating the effect on lipids (including lipoprotein(a)) and on ASCVD risk reduction. We also expanded the discussion on lipoprotein(a). Since we used isopycnic density gradient to isolate LDL fractions, we did not correct LDL-cholesterol for the cholesterol carried in lipoprotein(a). However, we mention that fractions 11-13 contain lipoprotein(a) and that this may impact on the cholesterol content of the smallest LDL subfraction.

Relative to ultracentrifugation, is it correct “48 hours at 158°C?”

Thank you for spotting this! The correct term is “48 hours at 15°C”. I guess, 158°C would not be very good for the lipoproteins!

Which statistical test has been used to evaluate differneces? My apology if I missed it.

We did not use any statistical tests, because we think that with n=3, statistical tests are not adequate. We have stated this in more clear way in the revised manuscript.

The impact of PCSK9 on triglycerides should be discussed in more details. Please refer to the review by Dijk W et al (PMID: 29665987) or Baragetti et al (PMID: 29428206).

In the revised version of the ms we discuss the effect of PCSK9 inhibition on TG in more detail, referring also to the reviews mentioned (thank you for providing these).

Which is the percentage of recurrence of FDBL? This could be reported both in the introduction and in the limitation section. Please report that it is a single centre study. The title should report that data have been obtained with evolocumab.

We have expanded the manuscript to include the topics raised by the reviewer. We also include evolocumab into the title, although we believe that the reported observations refer to a class effect. The new title is: “Effect of PCSK9 inhibition with evolocumab on lipoprotein subfractions in familial dysbetalipoproteinemia (type III hyperlipidemia)”.

**Reviewer #2:** In this study, the Authors investigated the impact of PCSK9 inhibition on lipoproteins in patients with FDBL. A comparison with patients with LDL-hypercholesterolemia and mixed hyperlipidemia has been made. PCSK9 inhibitors reduced cholesterol and apoB in all patients. In FDBL they specifically reduced VLDL-cholesterol. The Authors concluded that PCSK9-inhibition reduces the concentration of a wide variety of apoB-containing lipoproteins with the dominant particle size being reduced the most.

The study is original and interesting, however the paper is only descriptive, since sample size is too small and statistical analysis could not be performed.
This reviewer is aware of the rareness of familial dysbetalipoproteinemia, however three patients are not so much to draw robust conclusions, mostly because of the huge variability in lipid and lipoprotein profile.
Thus, more efforts should be dedicated to enroll additional subjects and increase sample size.

We thank the reviewer for the thoughtful and appreciative comments. It is true that the validity of our results is strongly limited by the small subject number. Unfortunately however, we do not have another subject with FDBL on PCSK9 inhibition in our registry. We therefore could not increase the sample size. On the other hand, as pointed out by the reviewer the variability in the lipid/lipoprotein profile of subjects with FDBL is huge, therefore one or 2 more subjects would most likely not add to clarity. At the same time the results in the three subjects were very consistent, which makes us confident that the effect is as described at least in subjects showing the typical lipid pattern.

Other comments:
Tables: how are data reported? Please provide whether the number are mean ± SD or median ± SEM.

Data are given as mean ± SD , which is now clearly stated in the revised manuscript.

Table 1: which is the prevalence of metabolic syndrome in these patients.

Prevalence of metabolic syndrome was added to the table.

Figure 2 is confusing. I would suggest using 3 different panels.

Thank you for your comment. We have not divided the figure into 3 panels, because we think having it in one figure allows for direct comparisons. To make the figure easier to read we have adjusted the labelling. However, if the reviewer feels strongly about dividing the figure into 3 different panels, we would be willing to do so.

Non si reduce solo il picco più alto. Si riduce tutto in proporzione

In the discussion section, we have clarified that all fractions are reduced in proportion.

State which inhibitor has been used also in method section, since it is reported only in the abstract.

Thank you for spotting this; we have added this information also to the method section. In addition, we have also included “evolocumab” to the title as requested by reviewer #1.

**Reviewer #3:** The study is interesting but some consideration has to be done.

We thank the reviewer for the constructive and helpful comments.

Please consider revising the description of the disease.

We have described the disease FDBL in more detail.

Aim. From your introduction, I do not understand what you want to test by comparing these population. Which differences do you expect? Why this study is relevant?

The indication for PCSK9 inhibitor therapy is usually based on the combined finding of a high/very high ASCVD risk with an LDL-cholesterol level, which is distant to the LDL-cholesterol goal. Although this combination is typical for FH, it can also be encountered in subjects with other underlying lipid disorders. We therefore evaluated the effect of PCSK9 inhibition with evolocumab on the whole spectrum of apoB containing lipoproteins in subjects who all had an indication for PCSK9 inhibition (according to German regulations) but were characterized by different underlying lipid disorders. We have clarified this in the revised version of the manuscript.

LDL-hypercholesterolemia, I imagine that these patients are heterozygous FH. Please use the correct terminology.

Although some of these patients have FH, some have other forms of primary LDL-hypercholesterolemia; we have clarified this in the revised version of the manuscript.

I do not understand the population selection:
1) Please do not give in methods any information on the demographic or clinical characteristics of these patients. These are reported in Table 1.

We have revised the method section, such that all subject information is now given in table 1/results

2)Did you genotype your patients? Both ApoE2/E2 patients and HeFH have already received a molecular confirmation? If yes, was it done for scientific purpose for this study or for clinical reasons?

No genotyping was performed; we use iso-electro focusing to determine the apoE phenotype; all patients were included in our registry on PCSK9 inhibition; the phenotyping was done for all patients included in the registry.

3)Nevertheless, how did you choose patients with mixed dyslipidemia and HeFH to be compared with FDBL. Did you take all your patients taking PCKS9i?

We selected consecutive patients with mixed hyperlipidemia and hypercholesterolemia to be compared to patients with FDBL. This was clarified in the revised version of the ms.

“All had the indication for treatment with PCSK9-inhibitors according to German
regulatory standards (not reaching treatment goals recommended by the European guidelines despite maximally tolerated oral lipid-lowering therapy)” please give a reference otherwise it is difficult to understand patient’s characteristics.

The reference was added.

Where PCKS9i given in a study protocol? I do not understand the study design. I guess if it is an observational real-world study or an interventional trial.

Patients were extracted from our registry on patients receiving PCSK9 inhibition. When PCSK9 inhibitors were available on the German market we started a registry in which patients treated in our center could be included (if they agreed and signed informed consent). This is clarified in the revised manuscript.

As you assess only lipoprotein fraction at baseline and 3 months a suggest you consider in your discussion that your results might be in “acute” phase of treatment.

Most data indicate that a new steady state with respect to lipid levels is achieved after 4-6 weeks of introducing/altering lipid lowering therapy. We therefore think that all values drawn after 3 months represent stable values that are unlikely to further change (if no change in therapy occurs).

Add significance to Table 1 (BMI is significantly different between groups)

We did not use any statistical tests, because we think that with n=3 in the FDBL group, statistical tests are not adequate. We have stated this in more clear way in the revised manuscript.

Please define CVD, CAD and PVD. Did you consider only acute events or all the events?

Any form of CVD, CAD and PVD was labelled as such, not only acute events. We have clarified this in the revised version of the ms.

Please also give statistics and the methodology used to analyze the data (reporting it in a separate chapter. Inferential statistics is completely lacking. You should add it throughout the manuscript (including Table 2 and figures).

As outlined above, we did not use any statistical tests, because we think that with n=3, statistical tests are not adequate. We have stated this in more clear way in the revised manuscript.

Moreover, there was any changes in the background lipid lowering therapies in the 3 months of treatment? Please add lipid lowering therapies to table 2.

No, there was no change in background therapy for any of the subjects included in the analysis. We have clarified this in the revised version of the manuscript. We have also added background lipid lowering therapy to the table 1.
